# Serum Cytokine Levels and Heart Rate Variability in the Frequency Domain in Patients With Chronic Chagas Heart Disease

**DOI:** 10.7759/cureus.99493

**Published:** 2025-12-17

**Authors:** Reinaldo B Bestetti, Renata Dellalibera-Joviliano, Milton Faria Junior, Rosemary A Furlan Daniel, Cláudia C Domingos

**Affiliations:** 1 Department of Medicine, University of Ribeirão Preto, Ribeirão Preto, BRA

**Keywords:** chagas disease, cytokines, frequency domain, heart rate variability, trypanosoma cruzi

## Abstract

The serum cytokine profile is well established in patients with chronic Chagas heart disease (CCHD). Little is known, however, about the relationship between plasma cytokine levels and frequency-domain parameters of heart rate variability (HRV) in patients with CCHD. We included patients with CCHD with ECG and/or echocardiographic abnormalities; people with no cardiac history matched by sex and age served as controls. ELISA performed cytokine quantification. The HRV in the frequency domain was used to detect parasympathetic and sympathetic dysfunction. Serum cytokine levels were higher in patients with CCHD than in controls. No correlation was found between TNF-α, IFN-γ, IL-1β, IL-4, IL-5, IL-6, IL-10, IL-12, IL-13, IL-8, IL-2, IL-7, TGF-β, IL-17, and HRV indices. However, a negative correlation between serum IL-23 levels and HRV indices in the high-frequency component was observed (p = 0.044; rho = -0.587). Whether IL-23 may play a role in the pathogenesis of CHCD remains to be determined.

## Introduction

In Latin America, about six million people are carriers of Chagas disease, and almost 70 million people are at risk of acquiring the disease. The illness is caused by the protozoan *Trypanosoma cruzi* (*T. cruzi*), which is transmitted to humans through the feces of a kissing bug [1]. Because of international immigration, Chagas disease has spread throughout the world; about 400,000 infected people have been estimated to live in non-endemic countries, mainly in the United States and Europe [2].

Infection usually occurs in early infancy; most patients recover completely. However, about 20% of patients will progress to chronic Chagas heart disease (CCHD) up to 20 years following infection [1]. Clinical manifestations of CCHD include chronic heart failure with reduced left ventricular ejection fraction (HFrEF) [3], thromboembolism [4], sudden cardiac death, advanced AV and fascicular blocks [5], ventricular arrhythmias [6], and precordial chest pain [7].

We and others have previously shown that patients with CCHD have elevated serum cytokine levels compared with normal subjects [8-11]. It is well known that high levels of cytokines may affect intracardiac autonomic function, the so-called inflammatory reflex [12]. Heart rate variability (HRV) is a phenomenon characterized by oscillations in the intervals between consecutive heartbeats and in the instantaneous heart rates. HRV in the frequency domain is a well-known method to measure parasympathetic and sympathetic activity [13]. Nevertheless, the impact of serum cytokine levels on HRV has been studied only sparingly in patients with CCHD [14]. Therefore, further studies are needed to assess any association between intracardiac autonomic function and myocardial inflammation parameters in patients with this condition.

Accordingly, the purpose of this study was to assess HRV in the frequency domain in relation to serum cytokine levels that mediate proinflammatory, anti-inflammatory, and regulatory activities in patients with CCHD to understand the impact of these cytokines on autonomic dysfunction in this condition.

## Materials and methods

Patients

The study has a cross-sectional design. Patients with positive serology for Chagas disease and an abnormal 12-lead ECG and/or an abnormal echocardiogram (interpreted according to standard criteria) were considered to have CCHD and were included in the study. Patients with only a positive serology for Chagas disease with no evidence of heart disease were ruled out of the investigation. In addition, patients with any other disease that could independently cause heart disease were excluded from the study. We used a convenience sample because of the low availability of patients at the time of cytokine measurement and the HRV in the frequency domain assessment.

Twelve patients with CCHD and eight control subjects with a negative serology, paired by sex and age, were included in the investigation. Patients with a left ventricular ejection fraction <55% on echocardiography were considered to have left ventricular systolic dysfunction (LVSD) and were treated with angiotensin-converting enzyme inhibitors or angiotensin receptor blockers, mineralocorticoid antagonists (spironolactone), and β-blockers at targeted doses or at the maximal tolerated dose, irrespective of the presence or absence of symptoms.

Patients with concomitant systemic arterial hypertension and no LVSD were treated at the discretion of the treating physician. In this study, we used patients as exclusion criteria: patients with known previous cardiovascular diseases with chronic or infectious-contagious evolution; patients undergoing previous cardiac surgery; presence of clinical or laboratory atherosclerosis; presence of autoimmune diseases; history of smoking.

The research project was approved by the Ethical Committee of the University of Ribeirão Preto (approval number: 48541715.5.0000.5498). It is reported that the data were collected from the medical records of the patients selected in this study.

We measured serum levels of the following cytokines in patients with CCHD and in controls using a double-ligand ELISA: TNF-α, IFN-γ, IL-1β, IL-4, IL-5, IL-6, IL-10, IL-12, IL-13, IL-8, IL-2, IL-7, TGF-β, IL-17, and IL-23, as previously described [15]. Briefly, the protocol used 96-well flat-bottom microtiter plates coated with 100 μL/well of specific antibody (Pharmingen, Saint Louis, MO) for one of the cytokines mentioned above, at an average concentration of 2 μg/mL in coating buffer, and incubated overnight at 4°C. The plates were then washed with the appropriate buffer and incubated for two hours at 37°C with buffer containing 1% bovine serum to prevent nonspecific binding (blocking step). Standard curves were constructed using the corresponding recombinant human cytokines. Samples and standards were placed in the wells and incubated overnight at 4°C. After careful washing, the appropriate biotinylated polyclonal or monoclonal anti-cytokine antibody was added. Following a one-hour incubation, the plates were washed three times with the appropriate buffer, and HRP-conjugated streptavidin (diluted 1:5000) was added. The plates were then incubated for 15 minutes and rewashed. Finally, the chromogenic substrate o-phenylenediamine (0.4 mg OPD + 0.4 μL H₂O₂ per 1 mL substrate buffer) was added, and the reaction was stopped 15 minutes later with 1 M H₂SO₄. The intensity of the developed color was measured spectrophotometrically at 490 nm using an ELISA plate reader. Serum cytokine levels are expressed as pg of cytokine per mL of the patients' serum.

HRV in the frequency domain was used to detect parasympathetic and sympathetic dysfunction. We use a commercially available digitized three-channel tape apparatus (Cardius Smart CS 550, Model 5.0382104) to perform HRV-FD. The duration of the heart rate register was 24 hours. The hourly low-frequency (LF) component and the high-frequency (HF) component were recorded. The sum of the LF component values was divided by 24 to obtain the mean. The same procedure was used to determine the mean value of the HF component. Patients with an LF/HF <1.5 were considered to have sympathetic derangement, whereas patients with an HF <700 Hz were diagnosed with parasympathetic impairment, as previously described [16]. It is reported that the data were collected from the medical records of the patients selected in this study.

Statistical analysis

Continuous variables are shown as mean ± standard deviation, whereas categorical variables are given as numbers (percentage). Serum cytokine levels between controls and CCHD patients were compared using the Mann-Whitney U test. Given the 15 independent cytokine comparisons, p-values were adjusted using the Bonferroni method (α = 0.05/15 = 0.00333). A result was considered statistically significant if the p-value according to the Bonferroni test was <0.00333. To reduce type I error due to multiple hypothesis testing, the Bonferroni correction was applied to clinically relevant cytokines selected a priori.

The correlation between the HF and LF/HF components of the HRV in the frequency domain and serum cytokine levels was assessed using Spearman's rank correlation. In all circumstances, a p < 0.05 value was considered to indicate statistical significance.

## Results

In patients with CCHD, the mean age was 69 ± 11 years; six (50%) were male. ECG changes were observed in six (50%) patients. Echocardiographic abnormalities were detected in all patients: three (25%) had left ventricular diastolic dysfunction, and nine (75%) had LVSD. Serum cytokine levels were higher in patients with CCHD than in controls. Raw p-values were subsequently adjusted for multiple testing using the Bonferroni method (α = 0.05/15 = 0.00333). As shown in Table 1, the adjusted p-value is below the Bonferroni threshold, indicating that the differences remain statistically significant.

**Table 1:** Comparison of cytokines serum levels (pg/ml) between controls and patients with CCHD * CCHD: chronic Chagas heart disease

Cytokines	COntrol	CCHD	U (value)	Bonferroni p-value	p-value
INF-γ	58 ± 1.5 pg/ml	124 ± 2.62 pg/ml	U = 0.0	0.00024	p < 0.0001
IL-2	27 ± 1.2 pg/ml	76 ± 2.2* pg/ml	U = 0.5	0.00027	p < 0.0001
IL-12	65 ± 1.4 pg/ml	289 ± 2.3* pg/ml	U = 0.0	0.00024	p < 0.0001
IL-4	40 ± 1.3 pg/ml	95 ± 2.6* pg/ml	U = 0.0	0.00024	p < 0.0001
IL-10	148 ± 2.7 pg/ml	275 ± 2.2* pg/ml	U = 0.5	0.00027	p < 0.0001
IL-13	46 ± 1.4 pg/ml	93 ± 1.8* pg/ml	U = 15.0	0.00023	p = 0.0004
IL-5	51 ± 2.4 pg/ml	207 ± 2.7* pg/ml	U = 0.0	0.00024	p < 0.0001
IL-17	75 ± 1.4 pg/ml	190 ± 2.1* pg/ml	U = 11.5	0.00024	p = 0.0002
IL-23	50 ± 1.6 pg/ml	170 ± 2.3* pg/ml	U = 0.0	0.00028	p < 0.0001
IL-6	78 ± 1.7 pg/ml	185 ± 2.2* pg/ml	U = 0.0	0.00023	p < 0.0001
TGF-β	31 ± 1.5 pg/ml	74 ± 1.3* pg/ml	U = 0.0	0.00027	p < 0.0001
TNF-α	225 ± 1.3 pg/ml	470 ± 3.2* pg/ml	U = 0.5	0.00024	p < 0.0001
IL-1β	51 ± 1.3 pg/ml	173 ± 1.5* pg/ml	U = 0.0	0.00594	p < 0.0001
IL-8	27 ± 2.7 pg/ml	54 ± 2.1* pg/ml	U = 0.0	0.00266	p < 0.0001
IL-7	1.9 ± 0.3 pg/ml	2.8 ± 0.5* pg/ml	U = 1.0	0.00024	p < 0.0001

**Figure 1:**
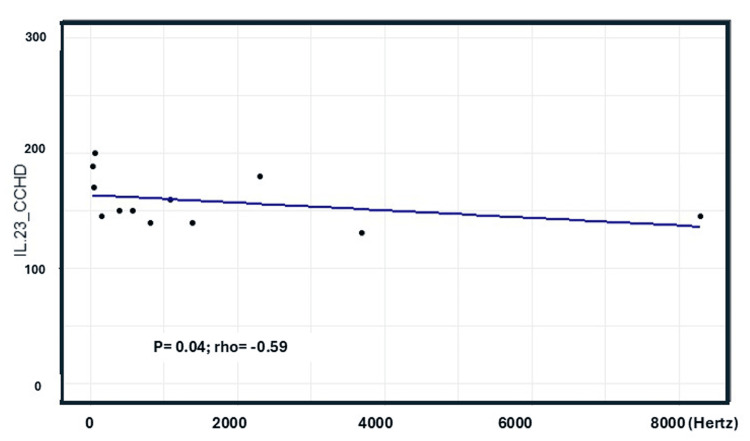
Correlation between IL-23 serum levels with the HF component of HRV in the frequency domain in patients with CCHD HF: high-frequency, HRV: heart rate variability, CCHD: chronic Chagas heart disease

No correlation was found between TNF-α, IFN-γ, IL-1β, IL-4, IL-5, IL-6, IL-10, IL-12, IL-13, IL-8, IL-2, IL-7, TGF-β, IL-17, and HRV indices. However, a negative moderate correlation between serum IL-23 levels and parameters of the HF component of the HRV in the frequency domain was observed (p = 0.044; rho = -0.587), as shown in Figure 1.

## Discussion

In this investigation, among several cytokines that express Th1, Th2, and regulatory patterns, we found a negative correlation between the IL-23 cytokine (a proinflammatory cytokine) and the HF component in the frequency domain of HRV. Th1 cells are associated with the destruction of intracellular pathogens and with autoimmune responses. Th2 cells play an essential role in controlling extracellular parasites and are linked to allergic diseases. Th17 cells are involved in the control of bacterial and fungal infections, are associated with autoimmune diseases, and induce the production of proinflammatory cytokines such as IL-6, IL-21, TGF-β, and particularly IL-23 [17]. To our knowledge, the correlation between an ample cytokine profile and parameters of autonomic activity, as detected by HRV in the frequency domain, has not previously been reported in patients with CCHD.

IL-23 is a proinflammatory cytokine belonging to the IL-12 family and is involved in the activation of 2Th17 proinflammatory cells. IL-23 has been found in the skin of patients with psoriasis, in the wall of patients with inflammatory bowel disease, and in the synovial membrane of patients with rheumatoid arthritis [18]. Therefore, IL-23 appears to be related to autoinflammatory diseases. Consequently, IL-23 blockade has been a target for treating patients with autoimmune diseases [19].

The immune response plays a crucial role in both the containment of *T. cruzi* and in the pathogenesis of CCHD. Experimental studies have shown that IL-23 is involved in the balance between protective immunity and immunopathology in *T. cruzi* infection. During acute *T. cruzi* infection, IL-23 is induced early, promoting the expansion of Th17 cells and the production of IL-17, which contribute to neutrophil recruitment and parasite containment [20].

On the other hand, the persistence of IL-23 may favor the maintenance of a chronic inflammatory response, especially in the myocardium, involving T-cell infiltration and the production of proinflammatory cytokines that exacerbate tissue damage, thereby leading to CCHD [21-22]. In fact, an experimental study showed that inhibition of IL-23 or IL-17 reduced cardiac inflammation and fibrosis, suggesting that the IL-23/Th17 axis may be a potential therapeutic target in patients with CCHD [19].

The negative correlation between serum IL-23 levels and the HF component of the HRV in the frequency domain may suggest a role for the anticholinergic pathway in the pathogenesis of CCHD. Several lines of evidence have supported a role for the parasympathetic nervous system impairment in the pathogenesis of CCHD. Parasympathetic dysfunction, along with autoimmunity and microvascular disease, is believed to play an essential role in the pathogenesis of CCHD [21]. Myocardial lesions in experimental *T. cruzi* infections are similar to those found in a catecholamine cardiomyopathy rat model [22]. β-adrenoceptor antagonists can mitigate the effects of *T. cruzi* infection in a rat model of CCHD [23]. In patients with HFrEF secondary to CCHD, coronary sinus adrenaline levels are increased in comparison with those with non-Chagas disease HFrEF, thus suggesting an intracardiac autonomic imbalance in Chagas disease patients [24].

Taken together, these facts may suggest that changes in parasympathetic function induced by elevated serum IL-23 levels may perpetuate myocardial inflammation and fibrosis, thereby contributing to left ventricular remodeling in patients with CCHD. On the other hand, it is essential to emphasize that serum levels of IFN-γ, IL-8, and TNF-α, cytokines implicated in the pathogenesis of CCHD [5], were not associated with autonomic dysfunction in this investigation.

The main weakness of this investigation is the small sample size. Another limitation is its cross-sectional design, which does not allow us to infer the impact of IL-23 over time. In addition, we did not provide mechanistic insights into the relationship between IL-23 and parasympathetic derangement, which warrants further study. Finally, our inability to control for the medications used did not allow us to assess their impact on autonomic activity. However, the correlation between IL-23 levels and parasympathetic system parameters, as measured by the HF component of HRV, was moderate. Therefore, despite the aforementioned caveats, we believe it is unlikely that our findings occurred by chance alone.

## Conclusions

Our study shows that serum IL-23 levels are inversely associated with parasympathetic function, as measured by HRV in the frequency domain, in patients with CCHD. However, the study design does not allow for causal inference between IL-23 and parasympathetic derangement. Whether IL-23 may play a role in the pathogenesis of CCHD remains to be determined.
